# Sleep Patterns and Influencing Factors in Romanian Medical Students During the COVID-19 Pandemic: A Two-Wave Ecological Study

**DOI:** 10.7759/cureus.51187

**Published:** 2023-12-27

**Authors:** Maria-Flavia Susanu, Raluca-Monica Pop

**Affiliations:** 1 Research Methodology Department, George Emil Palade University of Medicine, Pharmacy, Science and Technology of Târgu Mureș, Târgu Mureș, ROU; 2 Endocrinology Department, Mures County Emergency Clinical Hospital, Târgu Mureș, ROU; 3 Endocrinology Department, George Emil Palade University of Medicine, Pharmacy, Science and Technology of Târgu Mureș, Târgu Mureș, ROU

**Keywords:** covid-19, medical students, pandemic, sleep quality, screen time

## Abstract

Introduction

Sleep quality among students is variable with sleep disturbances being common worldwide. The coronavirus disease 2019 (COVID-19) pandemic led to major changes, including in the educational system. In this study, we aimed to analyze sleep patterns and screen time of medical students, and the effect of COVID-19.

Material and methods

We conducted a two-wave questionnaire-based ecological study on Romanian medical students from Târgu Mureș. For data collection, a 43-item questionnaire, structured into six categories, was designed and distributed through social networks and official online teaching platforms.

Results

Out of 751 answers from both waves, it was seen that most of the responders were female (76.23%, n=571) and in their preclinic years of study (61.33%, n=460). There was a statistically significant association between the form of education and students’ general sleep quality (p=0.0010, OR=1.670, 95%CI: 1.228-2.271), their study time (5.5 hours, IQR: 4-7.5 versus five hours, IQR: 2.5-5, p<0.001) and their sleep disturbances (p=0.0008, OR=0.5859, 95%CI: 0.4284-0.8011). Also, there was a statistically significant association between the year of study and their study time (five hours, IQR: 4-7 versus four hours, IQR: 3-6, p<0.001) and their sleep satisfaction (p=0.0027, OR=0.6360, 95%CI: 0.4729-0.8554).

Conclusions

Students reported better general sleep quality, less trouble sleeping, and less study while studying online full-time. Also, students in clinical years tended to study less and be more satisfied with their sleep compared to students in preclinical years.

## Introduction

Coronavirus disease 2019 (COVID-19) pandemic

The severe acute respiratory syndrome coronavirus 2 (SARS‑CoV‑2) virus started spreading from Asia to other continents all over the globe in November 2019 [1]. The World Health Organization (WHO) declared a Public Health Emergency of International Concern (PHEIC) on January 30, 2020, and a global pandemic on March 11, 2020, and this led to major changes in many areas [2,3]. In Romania, on March 16, 2020, an emergency state was declared by the President, and on March 25, 2020, a military curfew was instituted, which was lifted on May 15, 2020. After that, a state of alert was active country-wide with local lockdowns instituted for short periods depending on the incidence rate, which also lifted March 8, 2022. Regarding the educational system, all face-to-face education procedures were canceled for the second semester of the school year (March-June 2020) and universities relied mainly on online lectures and seminars, with medical universities canceling clinical rotation or restricting access and using a hybrid approach (online lectures and onsite practical activities).

Sleep disturbances

Sleep disturbances are prevalent globally, particularly among students, particularly medical students [4,5]. The COVID-19 pandemic further exacerbated sleep issues, as students faced challenges like lack of social interaction, lifestyle changes, and modified teaching-learning formats [6]. These factors may have significantly impacted their sleep quality, making them vulnerable to sleep disturbances.

The American Sleep Association states that young individuals aged 18-25 need seven to nine hours of sleep daily [7]. However, school and work schedules can disrupt these needs, leading to "social jetlag" [8]. Most people experience this, resulting in alarm clocks on workdays, sleep medication at night, and daytime stimulants to align sleep and wake times with social times [9].

Sleep deficit in adults is linked to cognitive impairment [10], metabolic disorders [11], and cardiovascular disease [12]. Medical students often experience sleep disturbances and dysfunction, and chronic stress can negatively impact sleep [5]. The COVID-19 pandemic has been identified as a risk factor for sleep disturbances [13].

Study objective

The aim of the current study was to examine screen time, sleep patterns, and the effects of online teaching on medical students during different periods of the COVID-19 pandemic.

## Materials and methods

Study design and participants

A two-wave questionnaire-based ecological study was conducted including medical students from Târgu Mureș, Romania. For the first wave, the questionnaire was distributed through social networks and the official online teaching-learning platform from October 16, 2020, to December 15, 2020, with online-only lectures. For the second wave, the questionnaire was distributed through social networks from March 9-30, 2022, when restrictions were partially lifted and hybrid classes were introduced.

To be included in the study, students had to be full-time students at the Faculty of Medicine of the George Emil Palade University of Medicine, Pharmacy, Science and Technology of Târgu Mureș (UMPhST), regardless of the teaching language. The study analyzed both complete and partial responses, with part-time students being excluded.

Survey instrumentation and distribution

For data collection, a 43-item questionnaire, structured into six categories (See Appendix), was designed and distributed online, containing questions about demographic characteristics, sleep patterns, sleep disturbances, daily activities, general questions about studying hours, sleep quality, and screen time, and specific, subjective questions about screen time. The questionnaire was entirely anonymous and, by returning the completed questionnaire, the students gave their informed consent for the processing of the data.

Social jetlag assessment

Regarding social jetlag, two parameters were tracked, namely social jetlag obtained from calculated values of students' sleep time and social jetlag obtained from students' self-reported sleep time. We will refer to these two parameters as calculated social jetlag and reported social jetlag, respectively, with the note that both values were obtained through a mathematical calculation, subtracting from the sleep time on nights preceding days off, the sleep time on nights preceding school days, to approximate social jetlag.

Data analysis

For data collection, Microsoft Excel (Microsoft Corporation, Redmond, Washington, United States) was used. The statistical analysis was performed using IBM SPSS Statistics for Windows, Version 25.0 (IBM Corp., Armonk, New York, United States), with the following statistical tests: Kolmogorov-Smirnov normality test, the Mann-Whitney test and the Kruskal-Wallis test for central tendencies comparison, Pearson Chi-Square coefficient for associations, Likelihood Ratio and Kendall's tau coefficient, for assessing the strength and direction of correlations between two variables. Qualitative variables were expressed as frequencies and percentages, while quantitative variables were expressed as mean ± standard deviation, median, and interquartile range (IQR), respectively. The confidence level was 95% and the p-value ≤0.05 was considered statistically significant.

Ethical approval and informed consent

The study was conducted in accordance with the Declaration of Helsinki, and approved by the Ethics Committee of the George Emil Palade University of Medicine, Pharmacy, Science and Technology of Tirgu-Mures (approval number: 1148/15.10.2020 for the first wave; approval number: 1625/24.02.2022 for the second wave). The students were informed that participation was anonymous and voluntary and by completing the questionnaire, they agreed that the data collected could be processed, on condition of anonymity.

## Results

Demographic characteristics of the study sample

Response Collection

In total, 751 completed questionnaires from both waves were considered for final analysis: 499 responses in the first wave and 252 responses in the second wave. Because the questionnaire was distributed only to medical students from UMPhST, it was possible to calculate the response rate and we obtained a total response rate of 18.57% for the first wave and 9.40% for the second wave, with variations over the study years, but with no statistically significant difference between groups regarding their sociodemographic characteristics.

Cohort Stratification

To analyze the distribution by years of study, we divided the students into two major groups: preclinical (from first year to third year of study) and clinical (from fourth to sixth year of study).

Demographic Profile

Regardless of the form of education, most students were females (76.23%, n=571), from urban areas (87.08%, n=654), and in their preclinical years of study (61.33%, n=460). The sample’s mean age was 22 years old (SD = 2, range = 18-28 years), with a BMI of 22.18 kg/m^2^ (SD=3.27, range=15.1-36 kg/m^2^).

Sociodemographic Consistency Across Waves

No statistically significant difference was observed between the two waves of the questionnaire in terms of the sociodemographic characteristics of the study group (all p>0.05).

Sleep patterns and daily activities

Sleep Duration Analysis

The second category of the questionnaire contained questions related to sleep patterns. Thus, on the nights preceding a school day, students slept 6.94 hours ± 1.05 hours (between 3.5 hours and 10 hours), considering the reported values; and 7.4 hours ± 1.17 hours (between 3.5 hours and 11 hours), considering the calculated. Also, on the nights preceding a day off, students sleep 8.64 hours ± 1.14 hours (between 3.5 hours and 12 hours), considering the reported values; and 8.73 hours ± 1.07 hours (between four hours and 13.5 hours), considering the calculated values. Both calculated and reported sleep time on nights before free days were unchanged regardless of the form of education (full online vs hybrid) or the preclinical/clinical year of study (all p>0.05). Also, both calculated and reported sleep time were unchanged regardless of the form of education (all p>0.05), but there was a statistically significant difference in the reported sleep time on nights before classes between the preclinical/clinical year of study (6.86±1.08 hours in preclinical vs 7.05±0.98 hours in clinical years of study, p=0.0138), but no significant difference in the calculated sleep time on nights before classes (p=0.3238).

Substance Use

In terms of students’ substance use habits, overall, it was observed that most students consumed coffee daily (70.84%, n=532), did not consume energy drinks (89.21%, n=670), did not use stimulant pills (82.69%, n=621), and did not use sleeping pills (87.62%, n=658). Moreover, the form of education didn’t influence the consumption of coffee, energy drinks, stimulant pills, or sleeping pills (all p>0.05). Additionally, the year of the study did not influence the consumption of coffee or sleeping pills (all p>0.05), but it did influence the consumption of energy drinks (p=0.001) and stimulant pills (p=0.003) (Table 1).

**Table 1:** Students’ habits regarding substance use * Chi-Square Test

Questionnaire items	Study wave (Form of education)		p-value	Preclinical/clinical year of study		p-value
	1^st^ wave (Full online)	2^nd^ wave (Hybrid)		Preclinical	Clinical	
	N (%)	N (%)		N (%)	N (%)	
Coffees per day						
0	150 (30.06)	69 (27.38)	0.764*	129 (27.98)	90 (31.03)	0.115*
1-2	307 (61.52)	158 (62.70)		282 (61.17)	183 (63.10)	
3-4	40 (8.02)	23 (9.13)		47 (10.20)	16 (5.52)	
5 or more	2 (0.40)	2 (0.79)		3 (0.65)	1 (0.34)	
Energy drinks per day						
0	447 (89.58)	223 (88.49)	0.196*	396 (85.90)	274 (94.48)	0.001*
1-2	48 (9.62)	23 (9.13)		56 (12.15)	15 (5.17)	
3-4	4 (0.80)	3 (1.19)		6 (1.30)	1 (0.34)	
5 or more	0 (0.00)	2 (0.79)		2 (0.43)	0 (0.00)	
Stimulant pills to help study/stay up						
No	415 (83.17)	206 (81.75)	0.814*	368 (79.83)	253 (87.24)	0.003*
Yes, over-the-counter stimulants (without prescription needed)	80 (16.03)	43 (17.06)		86 (18.66)	37 (12.76)	
Yes, prescribed stimulants	4 (0.80)	3 (1.19)		7 (1.52)	0 (0.00)	
Sleeping pills						
No	440 (88.35)	218 (86.51)	0.430*	408 (88.50)	250 (86.21)	0.510*
Yes, over-the-counter pills (without prescription needed)	50 (10.04)	31 (12.30)		46 (9.98)	35 (12.07)	
Yes, prescribed pills	8 (1.61)	2 (0.79)		5 (1.08)	5 (1.72)	

Daytime Fatigue and Sleepiness

Afterward, there were four questions related to daytime fatigue and sleepiness. Thus, a statistically significant association was observed between the preclinical/clinical year of study and feeling tired in the morning (p<0.001), feeling sleepy during daytime (p=0.002), and feeling sleepy during free time (p=0.001), but no significant association with feeling sleepy during lectures (p=0.213). On the other hand, there was no significant association between the form of education (full online vs hybrid approach) and feeling tired/sleepy in these four moments (all p>0.05) (Table 2).

**Table 2:** Daytime fatigue and drowsiness * Chi-Square Test

Questionnaire items	Study wave (Form of education)		p-value	Year of study		p-value
	1^st^ wave (Full online)	2^nd^ wave (Hybrid)		Preclinical	Clinical	
	N (%)	N (%)		N (%)	N (%)	
Tired in the morning						
Never	21 (4.21)	10 (3.97)	0.588*	17 (3.69)	14 (4.83)	<0.001*
Less than once a week	85 (17.03)	32 (12.70)		49 (10.63)	68 (23.45)	
1-2 days a week	140 (28.06)	79 (31.35)		130 (28.20)	89 (30.69)	
3-4 days a week	104 (20.84)	53 (21.03)		106 (22.99)	51 (17.59)	
Daily	144 (28.86)	77 (30.56)		155 (33.62)	66 (22.76)	
Sleepy during daytime						
Never	24 (4.81)	11 (4.37)	0.280*	20 (4.34)	15 (5.17)	0.002*
Less than once a week	81 (16.23)	26 (10.32)		49 (10.63)	58 (20.00)	
1-2 days a week	172 (34.47)	92 (36.51)		158 (34.27)	106 (36.55)	
3-4 days a week	111 (22.24)	62 (24.60)		114 (24.73)	59 (20.34)	
Daily	109 (21.84)	60 (23.81)		117 (25.38)	52 (17.93)	
Sleepy during lectures						
Never	30 (6.01)	14 (5.56)	0.384*	24 (5.21)	20 (6.90)	0.213*
Less than once a week	66 (13.23)	22 (8.73)		45 (9.76)	43 (14.83)	
1-2 days a week	153 (30.66)	89 (35.32)		153 (33.19)	89 (30.69)	
3-4 days a week	113 (22.65)	60 (23.81)		111 (24.08)	62 (21.38)	
Daily	134 (26.85)	67 (26.59)		126 (27.33)	75 (25.86)	
Sleepy during free time						
Never	72 (14.43)	36 (14.29)	0.494*	63 (13.67)	45 (15.52)	0.001*
Less than once a week	113 (22.65)	44 (17.46)		75 (16.27)	82 (28.28)	
1-2 days a week	168 (33.67)	89 (35.32)		167 (36.23)	90 (31.03)	
3-4 days a week	69 (13.83)	36 (14.29)		73 (15.84)	32 (11.03)	
Daily	75 (15.03)	46 (18.25)		81 (17.57)	40 (13.79)	

Study Time and Sleep Satisfaction

Analyzing the fifth category of the questionnaire, a statistically significant difference was observed in the median study time between the two waves (5.5 hours, IQR: 4-7.5 hours versus five hours, IQR: 2.5-5 hours, p<0.001) and between preclinical and clinical years of study (5 hours, IQR: 4-7 hours versus 4 hours, IQR: 3-6 hours, p<0.001). Also, there was no statistically significant association between the form of education and sleep satisfaction (p=0.0678, OR=1.328, 95%CI: 0.9790-1.801), but there was a statistically significant association between the preclinical/clinical year of study and the sleep satisfaction (p=0.0027, OR=0.6360, 95%CI: 0.4729-0.8554). When asked if they had trouble sleeping, there was a statistically significant association between the form of education and having trouble sleeping (p=0.0008, OR=0.5859, 95%CI: 0.4284-0.8011), but no significant association between the preclinical/clinical year of study and having trouble sleeping (p=0.6110, OR=1.083, 95%CI: 0.7958-1.475). In addition, most students reported poor sleep quality on nights before exams (43.40%, n=326), regardless of the form of education (p=0.1139) or the preclinical/clinical year of study (p=0.7288). Also, there was no statistically significant association between the general sleep quality and the preclinical/clinical year of study (p=0.0838), but there was a statistically significant association between the form of education and the general sleep quality (p=0.0033). When grouped in excellent-good sleep quality and satisfactory-poor sleep quality, there was a statistically significant positive association between these and the form of education (p=0.0010, OR=1.670, 95%CI: 1.228-2.271) (Table 3).

**Table 3:** Sleep disturbances * Chi-Square Test

Questionnaire items	Study wave (Form of education)		p-value	Preclinical/clinical year of study		p-value
	1^st^ wave (Full online)	2^nd^ wave (Hybrid)		Preclinical	Clinical	
	N (%)	N (%)		N (%)	N (%)	
Sleep satisfaction						
No	243 (48.70)	141 (55.95)	0.0678*	256 (55.53)	128 (44.14)	0.0027*
Yes	254 (50.90)	111 (44.05)		204 (44.25)	161 (55.52)	
Sleep disturbances						
No	338 (67.74)	140 (55.56)	0.0008*	291 (63.12)	187 (64.48)	0.6110*
Yes	157 (31.46)	111 (44.05)		168 (36.44)	100 (34.48)	
General sleep quality						
Excellent	64 (12.83)	25 (9.92)	0.0033*	47 (10.20)	42 (14.48)	0.0838*
Good	253 (50.70)	104 (41.27)		218 (47.29)	139 (47.93)	
Satisfactory	135 (24.05)	81 (32.14)		132 (28.63)	84 (28.97)	
Poor	46 (9.22)	42 (16.67)		63 (13.67)	25 (8.62)	
Sleep quality before exams						
Excellent	20 (4.01)	9 (3.57)	0.1139*	16 (3.47)	13 (4.48)	0.7288*
Good	119 (23.85)	53 (21.03)		101 (21.91)	71 (24.48)	
Satisfactory	158 (31.66)	65 (25.79)		138 (29.93)	85 (29.31)	
Poor	201 (40.28)	125 (49.60)		205 (44.47)	121 (41.72)	

Screen time

The final category of our questionnaire contained questions regarding students’ screen time. Accordingly, there was a statistically significant association between the study wave (form of education) and daily screen time (p<0.001), and between the preclinical/clinical year of study and daily screen time (p=0.038). Additionally, there was no significant association between the form of education and the use of devices right before going to bed (p=0.059), nor between the preclinical/clinical year of study and the use of devices right before going to bed (p=0.356) (Table 4).

**Table 4:** Screen time * Chi-Square Test

Questionnaire items	Study wave (Form of education)		p-value	Preclinical/clinical year of study		p-value
	1^st^ wave (Full online)	2^nd^ wave (Hybrid)		Preclinical	Clinical	
	N (%)	N (%)		N (%)	N (%)	
Screen time (hours/day)						
0-1	5 (1.00)	10 (3.97)	<0.001*	13 (2.82)	2 (0.69)	0.038*
1-3	103 (20.64)	49 (19.44)		90 (19.52)	62 (21.38)	
3-5	131 (26.25)	109 (43.25)		135 (29.28)	105 (36.21)	
5 or more	257 (51.50)	84 (33.33)		220 (47.72)	121 (41.72)	
Devices right before going to bed						
Never	4 (0.80)	1 (0.40)	0.059*	3 (0.65)	2 (0.69)	0.356*
Less than once a week	6 (1.20)	10 (3.97)		9 (1.95)	7 (2.41)	
1-2 days a week	25 (5.01)	18 (7.14)		29 (6.29)	14 (4.83)	
3-4 days a week	48 (9.62)	30 (11.90)		55 (11.93)	23 (7.93)	
Daily	414 (82.97)	192 (76.19)		362 (78.52)	244 (84.14)	

Social jetlag

Social Jetlag Assessment

In addition, special attention was also paid to the notion of social jetlag. Thus, there was no statistically significant difference in calculated social jetlag between the two waves, gender and background, nor between preclinical/clinical years of study (all p>0.05). Also, in terms of reported social jetlag, there was a statistically significant difference between the two waves (p=0.0292) and between preclinical/clinical years of study (p=0.0047), but no difference in social jetlag between gender nor by background (all p>0.05) (Table 5).

**Table 5:** Social jetlag * Mann-Whitney U test

Social jetlag	Total	Study wave (Form of education)		p-value	Preclinical/clinical year of study		p-value
		1^st^ wave (Full online)	2^nd^ wave (Hybrid)		Preclinical	Clinical	
Calculated social jetlag	1 hour, IQR: 0.5-2 hours	1 hour, IQR: 0.5-2 hours	1 hour, IQR: 0.5-2 hours	0.8847*	1 hour, IQR: 0.5-2 hours	1 hour, IQR: 0.5-2 hours	0.1055*
Reported social jetlag	1.5 hours, IQR: 1-2.5 hours	1.5 hours, IQR: 1-2.5 hours	2 hours, IQR: 1-2.5 hours	0.0292*	2 hours, IQR: 1-2.5 hours	1.5 hours, IQR: 1-2 hours	0.0047*

Social Jetlag Categories and Associations

Students were grouped into two categories: with social jetlag ≥ 1 hour and with social jetlag < 1 hour. It was observed that, overall, most students exhibited social jetlag ≥ 1 hour, considering both calculated social jetlag (63.91%, n=480) and reported social jetlag (79.76%, n=599). A statistically significant negative association was observed between the form of education and reported social jetlag (p=0.0021, OR=0.5266, 95%CI: 0.3485-0.7958). However, no statistically significant association was observed between the form of education and calculated social jetlag (p=0.7555). Social jetlag was not influenced by student gender, background, or preclinical/clinical year of study (all p>0.05).

Associations with Sleep Satisfaction and Disturbances

In addition, a statistically significant positive association was observed between sleep satisfaction and calculated social jetlag (p=0.0011, OR=1.647, 95%CI: 1.219-2.225), as well as between reported social jetlag and sleep satisfaction (p<0.0001, OR=2.292, 95%CI: 1.582-3.322).

There was also a statistically significant positive association between reported social jetlag and having trouble sleeping (p=0.0098, OR=1.684, 95%CI: 1.131-2.507), but not between calculated social jetlag and having trouble sleeping (p=0.7946). Also, social jetlag did not have a significant influence on students’ sleep quality in general or on students’ sleep quality on nights before exams (all p>0.05). Also, neither the use of screen devices before bedtime nor the amount of time spent in front of a screen influenced social jetlag (all p>0.05).

## Discussion

The study aimed to examine sleep patterns and screen time habits among UMPhST medical students and the impact of COVID-19 on their sleep. Results showed that students' sleep patterns and screen usage behavior were similar to current literature [14].

Contextual factors and educational changes

During the first wave of our study (October 16, 2020, to December 15, 2020), students participated in courses exclusively online. At that time, the number of confirmed COVID-19 cases was increasing and there was still no vaccine available [15]. There were not yet enough studies on SARS-CoV-2, but it was known that the people most severely affected were the elderly [16]. In Romania, the vaccination campaign started on December 27, 2020, with the vaccination of medical staff. Subsequently, the vaccination campaign was extended to the entire population [15]. The second wave of study took place in March 2022, when students participated in a hybrid learning format, with online courses and onsite practical work. The number of COVID-19-positive cases was already decreasing, with about half of the world's population already vaccinated [17].

The present study showed that the form of education influenced medical students’ general sleep quality, their screen time, their study time, and the fact of experiencing sleep disturbances. In our study, students tend to report a better general sleep quality, less trouble sleeping, and study less while studying online full-time, during the first wave of our study. Also, the form of education along with the pandemic situation didn’t influence their sleep time.

On the contrary, studies show a high prevalence of poor sleep quality among medical students during the COVID-19 pandemic, regardless of location [6,18]. This difference may be due to the shift to online courses and exams, which made students from our study feel more protected. The illusion of immortality, which is specific to young people, may also contribute to the issue [19].

Influence of academic year on sleep and substance use

In addition, in our study, the preclinical/clinical year of study had a great influence on the parameters analyzed. Thus, it was shown that students in clinical years tended to study less, be more satisfied with their sleep, and sleep more on nights preceding school days compared to students in preclinical years. Also, students in their preclinical years tended to consume more energy drinks and stimulant pills. Medical students in their preclinical years tended to be more stressed, regardless of the pandemic situation [20]. The transition from high school to medical school is abrupt and the workload increases exponentially. The academic pressure is high and they have to learn a lot within a limited time span [21].

Social jetlag insights

Regarding social jetlag, Foster et al. showed that sleep and wake cycles, duration, quality, and efficiency of sleep are influenced by social timing, and in turn, they influence mood, cognitive processing, and mental wellbeing [9]. In our study, social jetlag was around 1.5 hours, and most students experienced social jetlag ≥ 1 hour. The form of education influenced social jetlag, with students having lower social jetlag during online-only education. This could be explained by the fact that students no longer had to wake up in the morning to go to classes but only had to log in to specific platforms to attend classes. On the other hand, in our study, the preclinical/clinical year of study did not influence social jetlag. In addition, students with social jetlag ≥ 1 hour are more likely to be less satisfied with their sleep.

Urban vs. rural differences in sleep duration

Roenneberg et al. found that urban individuals sleep later and sleep less [22]. Our study found that sleep time (both reported and calculated) on nights preceding school days remains unchanged regardless of background, but sleep duration on nights preceding days off differs significantly. Thus, in our study, urban students sleep less than rural students on nights before school holidays.

Gender differences and sleep patterns

In terms of biological factors, our hypothesis was that women would have shorter sleep duration and higher social jetlag than men. This hypothesis was based on the literature that highlights an influence of external aspects, such as a double working day (work plus home: cleaning, children, etc.), which was associated with a shorter sleep duration in women relative to men [23]. However, our study did not show a significant gender difference in sleep duration, except for the reported sleep duration on nights preceding days off, nor in social jetlag. This could be because the sample was predominantly female and unmarried, resulting in no experience of a double workday, and the non-probability sampling method used, obtaining a convenience sample.

Response rate and limitations

Furthermore, it may seem like we obtained a quite low response rate, especially for the second wave (18.57% and 9.4%), but it is well known that much lower response rates are obtained through online distribution, compared to the physical distribution of questionnaires [24]. However, this could be still a low representation of the situation, which could impact our results. To increase response rates, we facilitated students' access to the questionnaire by giving them the survey webpage address on social media groups and the official online teaching-learning platform groups. We provided frequent reminders, approximately once every two to three weeks, with a maximum of four reminders, to avoid irritation of the target population and a possible refusal of participants to complete the questionnaire [24]. Teachers from UMPhST Târgu Mureș were involved in distributing the questionnaire through the official online platform, sending the webpage address to students at the beginning of courses or practical activities. However, students from all six years of study and all three lines of teaching were included in the study, which supports the diversity and variety of responses received and included in the study.

Potential biases and future directions

As a limitation of our study, it is mandatory to mention that a self-composed questionnaire was used for this study and only nine questions were adopted from the Pittsburgh Sleep Quality Index (PSQI). Even if many topics were covered, we didn’t use any validated questionnaire to assess sleep.

Our study did not consider other factors influencing medical students' sleep, such as socioeconomic status, and housing conditions. Lower socioeconomic status and less advantaged social classes tend to have greater sleep problems, leading to shorter and lower-quality sleep [25]. Housing conditions, such as overcrowded houses or sharing rooms, are also associated with poorer sleep quality [26].

Additionally, we must mention that students’ sleep problems could be much more serious than those reported in this study, as students may provide altered, socially desirable answers, such as reasons for falling asleep late, waking up or bedtime schedules, as well as screen time or pill intake [27]. At the same time, as the data were based on self-rating measurements, it might introduce recall bias.

Second, most of our questions were related to the week before receiving the questionnaire, which may or may not be representative of students' sleep habits, in general. Our questionnaire didn’t make any difference between the semester period and the exam session, in which it is well known that stress is much more evident in students' lives and in which both study time and time spent in front of screens are different from the semester period.

A future study could make a clearer difference between the devices and use various applications to better monitor screen time. At the same time, our study didn’t consider the media content that students access [28] or the screens' light spectrum, both of them being able to influence sleep quality [29].

Furthermore, this study included only medical UMPhST students, limiting the student population included in the study and the findings may not be generalized to other students. Moreover, living in a small city, like Târgu Mureș, can often lead to less stress in people compared to those in larger urban areas. Smaller cities typically offer a slower pace of life, lower levels of noise, and a stronger sense of community. These factors can contribute to reduced stress levels, as individuals may experience shorter commutes and a greater sense of connectedness with their neighbors, ultimately leading to a calmer and more relaxed lifestyle. Future studies could include medical students enrolled in other universities in the country, possibly comparing the results by university. Further studies may also include students from other faculties, not just medicine, to highlight any differences.

The pandemic's broader impact

Finally, differences in the sleep quality of the students could not be established as we did not have any baseline data from before the COVID-19 pandemic. Moreover, the COVID-19 pandemic could have been a major disruptor itself, which might have played a role in student’s sleep quality. In their study, Gotlib et al. observed that the development of the adolescent brain was greatly influenced by the COVID-19 pandemic, with accelerated brain maturation, with signs of advanced cortical thinning and larger bilateral hippocampal and amygdala volumes in peri-pandemic adolescents [30]. Thereby, the potential influence of the COVID-19 pandemic could be much bigger and not only reflected on students' sleep quality but also on their performance, their health (both mental and physical health), and their future patients’ security.

## Conclusions

Students reported better general sleep quality, less trouble sleeping, and less study while studying online full-time. Also, the form of education along with the pandemic situation didn’t influence their sleep time. Moreover, students in clinical years tended to study less, be more satisfied with their sleep, and slept more on nights preceding school days compared to students in preclinical years. Also, students in their preclinical years tended to consume more energy drinks and stimulant pills and experienced tiredness and fatigue.

## Appendices

The 43-item questionnaire

Questions 13, 14, 15, 16, 17, 18, 22, 23, and 25 are adopted from the Pittsburgh Sleep Quality Index (PSQI).

1.  How old are you?

................................................................

2.   What’s your biological sex?

a)    Male

b)    Female

3. At what faculty are you enrolled?

a) Medicine_LR (Romanian)

b) Medicine_LM (Hungarian)

c) Medicine_LE (English)

4. Year of study:

o   1

o   2

o   3

o   4

o   5

o   6

5. Please write your height (cm):

...............

6. Please write your weight (kg):

................

7. In which city do you study? (If you don't study in Târgu Mureș, please write in the "other" section the city where you study)

a) Târgu Mureș, Mureș, Romania

b)  Others......

8. Where are you from? (name of home town)

.......................................................................................................................

9. Do you live in an urban or a rural area?

a) Urban

b) Rural

10.  Do you have a day job? (If yes, please write how many hours/day you work in the "other" section)

a) Yes

b) No

c)  .... hours

11.  Do you have a night shift job? (If yes, please write how many hours/night you work in the "other" section)

a)    Yes

b)    No

c)     .... hours

12.  At what time do you usually go to bed on nights before classes?

o   21:00

o   21:30

o   22:00

o   22:30

o   23:00

o   23:30

o   00:00

o   00:30

o   01:00

o   01:30

o   02:00

o   02:20

o   03:00

o   03:30

13. At what time do you usually wake up on school days?

o   05:00

o   05:30

o   06:00

o   06:30

o   07:00

o   07:30

o   08:00

o   08:30

o   09:00

o   09:30

o   10:00

o   10:30

o   11:00

o   11:30

o   12:00

14.   How many hours of sleep do you usually have on nights before classes?

...............................................................................................................................

15.  At what time do you usually go to bed on nights without classes (ex: holidays, week-end etc)?

o   21:00

o   21:30

o   22:00

o   22:30

o   23:00

o   23:30

o   00:00

o   00:30

o   01:00

o   01:30

o   02:00

o   02:20

o   03:00

o   03:30

16. At what time do you usually wake up on free days (ex: holidays, week-end etc)?

o   05:00

o   05:30

o   06:00

o   06:30

o   07:00

o   07:30

o   08:00

o   08:30

o   09:00

o   09:30

o   10:00

o   10:30

o   11:00

o   11:30

o   12:00

17. How many hours of sleep do you usually have on nights without classes?

...............................................................................................................................

18.  How many coffees do you usually drink per day?

a)    0

b)    1-2

c)     3-4

d)    5 or more

19.  How many energy drinks do you usually drink per day?

a)    0

b)    1-2

c)     3-4

d)    5 or more

20.  Do you use stimulant pills to help you study/stay up late/work?

a)    No

b)    Yes, Over-the-Counter stimulants (without prescription needed)

c)     Yes, prescribed stimulants

21.   Do you use pills to help you sleep?

a)    No

b)    Yes, Over-the-Counter pills (without prescription needed)

c)     Yes, prescribed pills

22.   How long (in minutes) does it take for you to fall asleep each night?

a)    <15 min

b)    16-30 min

c)     31-60 min

d)    >60 min

23.  How many times do you wake up during your sleep?

a)    0

b)    1-2 times per night

c)     3-4 times per night

d)    More than 5 times per night

24. If yes, what are the reasons why you have trouble sleeping? (mark all that apply)

a)    Cannot get to sleep within 30 minutes

b)    Wake up in the middle of the night or early morning

c)     Have to get up to use the bathroom

d)    Cannot breathe comfortably

e)    Cough or snore loudly

f)      Feel too cold

g)    Feel too hot

h)    Have bad dreams

i)   Have pain

j)  Other reason(s)

25.  What do you usually do before going to bed?

a)    Read a book

b)    Use cell phone

c)     Use computer

d)    Do sports

e)    Others (specify): .......

26.  What are the reasons why you go to sleep late?

a)    Study

b)    Working / job

c)     Playing video games

d)    Socialize (ex: facebook, instagram, tinder etc)

e)    Others (specify): ...........

27.   Do you take any naps during the day?

a)    Yes

b)    No

28.  If "yes”, how many naps do you usually take during the day?

a)    1-2

b)    2-4

c)     More than 5

29.  How much time do you „nap” in total per day?

a)    <30 min

b)    30-60 min

c)     60-120 min

d)    More than 120 min

30.  During the last week, did you feel tired in the morning?

a)  Never

b)  Less than once a week

c)  1-2 days a week

d)  3-4 days a week

e)   Daily

31.  During the last week, did you feel sleepy during daytime?

a)   Never

b)  Less than once a week

c)  1-2 days a week

d)   3-4 days a week

e)  Daily

32.  During the last week, did you feel sleepy during the lectures?

a)  Never

b)  Less than once a week

c)  1-2 days a week

d)  3-4 days a week

e) Daily

33.  During the last week, did you feel sleepy during free time?

a)  Never

b)  Less than once a week

c)  1-2 days a week

d)  3-4 days a week

e)  Daily

34.  How many hours do you usually study per day?

...............................................................................................................................

35.   Do you consider yourself a morning person?

a)   Yes

b)   No

36.  Are you satisfied with your sleep?

a)   Yes

b)   No

37.  Do you have trouble sleeping?

a)  Yes

b)  No

38.  How would you evaluate your sleep quality?

a)  Excellent

b)   Good

c)   Satisfactory

d)  Poor

39. How would you evaluate your sleep quality on nights before an exam?

a)    Excellent

b)    Good

c)     Satisfactory

d)    Poor

40.  How many hours do you estimate you spend a day in front of a screen? (TV, phone, computer etc)

a)    0-1

b)    1-3

c)     3-5

d)    5 or more

41.   How soon after waiking up do you interact with a device?

a)    Within seconds

b)    After several minutes

c)     After several hours

42.   During the last week, did you use any device right before going to sleep?

a)    Never

b)    Less than once a week

c)     1-2 days a week

d)    3-4 days a week

e)    Daily

43.   For what purpose do you usually use “the screens”?

a)    TV viewing

b)    Computer games

c)     Internet for non-study reasons (hobbies)

d)    Internet for study reasons

THANK YOU FOR YOUR COOPERATION

Informed Consent

By completing this questionnaire, you agree that the above data will be processed - on condition of anonymity - in order to develop a study.
